# ADaPTS “(AD)olescents (P)ath through (T)ransplant (S)ickle cell disease”

**DOI:** 10.1186/s12955-022-02021-w

**Published:** 2022-07-30

**Authors:** Aisha A. K. Bruce, Gregory M. T. Guilcher, Sunil Desai, Tony H. Truong, Michael Leaker, Dominic A. Alaazi, Sasia J. V. Pedersen, Bukola Salami

**Affiliations:** 1 Division of Pediatric Hematology and Oncology, 3-467 Edmonton Clinic Health Academy (ECHA), Department of Pediatrics, Faculty of Medicine, University of Alberta, 11405 - 87 Avenue, Edmonton, AB T6G 1C9 Canada; 2 Stollery Children’s Hospital, Alberta Health Services, Edmonton, AB Canada; 3 Section of Pediatric Oncology/Cellular Therapy, Alberta Children’s Hospital, Departments of Oncology and Pediatrics, Cumming School of Medicine, Calgary, AB Canada; 4 Faculty of Nursing, University of Alberta, Edmonton, AB Canada

## Abstract

**Background:**

Sickle cell disease is an inherited chronic hematological disorder with an average lifespan of fifty years. The human cost of sickle cell disease includes missed school days, occupational opportunities, social isolation, stigmatization, and psychological sequelae. Hematopoietic cell transplantation (HCT) is the only curative therapy available but comes with potential morbidity and mortality. Our study explores how quality of life (QoL) is affected from the perspective of an adolescent who has undergone a nonmyeloablative matched sibling donor HCT.

**Methods:**

We employed multiple case study methodology with purposeful sampling by selecting information-rich cases. Data sources: 1) QoL inventories 2) patient interviews 3) parent interview 4) vital support interview 5) medical record analysis. Data analysis: Intra-case analysis by assembling evidence within a single case and then analyzing the differences within cases to create a rich case description. Next, a time series analysis was completed to track changes in patients’ QoL. We used multiple sources of data to compose a timeline and changes across time. Then, we employed pattern matching as an analytical technique allowing for examination of patterns across cases. Finally, we used cross case synthesis to review results of each case.

**Results:**

Quality of life was reported across the physical, social and psychological domains for 5 participants. All had sickle cell HgSS genotype, 80% were male and 80% were born outside of Canada. Physical domain: pre-transplant, 100% of patients experienced pain, and the majority suffered from fatigue, insomnia, and fevers resulting in hospitalizations. Afterwards, participants reported improved physical wellbeing. Social domain: pre-transplant, QoL was poor characterized by stigma, social isolation, and parental absenteeism. Post-HSCT adolescents gained social acceptance in areas that had stigmatized and excluded them. They were able to participate freely in activities with peers and their social life vastly improved. Psychological pre-transplant life experiences were overshadowed by psychological stress. The majority commented that their future was bleak and may lead to premature death. Afterwards adolescents described a crisis free life with positive psychological outcomes.

**Conclusions:**

Adolescents with sickle cell disease who undertook HCT demonstrated improved QoL one year post transplant with regard to physical, social and psychological well-being.

## Background

Sickle cell disease (SCD) affects millions worldwide and an estimated two million Americans carry the sickle cell allele [1]. The complications experienced by people with SCD can start in infancy and range from painful vaso-occlusive crises, stroke, acute chest episodes, life-threatening anemia, and early death with an estimated lifespan of 5 decades. In addition, the human cost of sickle cell disease includes missed school days, occupational opportunities, social isolation, stigmatization, and psychological strain [2–4]. Most parents of SCD patients cared for in Alberta have emigrated from Africa where the disease is perceived differently and often carries stigma, isolation and a much broader awareness of the implications of the diagnosis than in North America [5]. There has been a steady population rise (tripled in three years) with new immigrant families accounting for 60% of the pediatric population in Northern Alberta [6]. For families the concept of cure is embraced fervently as they watch their children suffer and have experienced devastating consequences of the disease as well as stigma from their community.

The lifetime costs for SCD has been estimated at 9 million US dollars due to the acute and chronic complications [7]. Thus, due to the high disease burden for the individual, shortened lifespan and significant costs to the patient, their family, and the medical system there is significant interest in curative therapy.

The advent of reduced-intensity conditioning (RIC) and non-myeloablative HCT regimens, a new alternative with a more acceptable toxicity profile, allows patients to contemplate curative therapy [8, 9]. This treatment has demonstrated high rates of engraftment with no graft vs. host disease (GVHD) and successfully reported pregnancies [10, 11]. Currently, non-myeloablative HCT is only available for patients with an ABO compatible (or minor incompatible) HLA-identical matched sibling donor, meaning fewer than 20% of patients have this curative option.

Alberta Children’s Hospital (ACH) has implemented the NIH protocol [10] for children with SCD with excellent success. To date, all patients have engrafted and none have experienced acute or chronic GVHD [12]. With implications for resources on an individual, family and healthcare level further knowledge from the patient’s perspective is needed. There is a dearth of published qualitative data exploring the experiences of SCD patients who have undergone HCT to inform practice.

There are quality-of-life (QoL) studies pre/post HCT in SCD patients with the majority examining myeloablative or reduced-toxicity HCT conditioning regimens. Bhatia et al. [13] examined QoL in recipients receiving myeloablative reduced-toxicity HCT for SCD and demonstrated an improved QoL in all domains other than the social domain 6 months and 1 year after transplant. While the study size was small the positive results were encouraging.

There is limited information on how the adolescent SCD transplant recipient views their QoL and whether it is improved after transplantation. Previous literature would suggest that patients who undergo myeloablative reduced-toxicity HCT for SCD have improved quality of life overall.

The objective of the study is to understand the changes in QoL for adolescent patients with sickle cell disease a year after transplant.

## Methods

### Study design

Multiple case study methodology. A case study is “an empirical enquiry that investigates a contemporary phenomenon within its real life context, when the boundaries between phenomenon and context are not clearly evident and in which multiple sources of evidence are used” [14]. The unit of analysis is the patient who has undergone a HCT to treat SCD. To fully understand the complexity of changes in QoL for SCD patients within a year after the hematopoietic stem cell transplant, the data was collected from multiple sources including patients, medical records, QoL inventories, parents, and vital networks.

### Sampling and recruitment

Patients were recruited from two sickle cell disease programs in Alberta, Canada. The study was approved by the University of Alberta Research Ethic’s Board. Verbal and written consent was obtained for all participants.

A purposeful sampling of 5 cases enables the selection of information rich cases [15, 16]. Yin identified that as few as 5 cases can serve as a large case size for a case study [16]. The inclusion criteria for the study: (1) age 13–18 years; (2) a medical diagnosis of SCD; (3) non-myeloablative HCT with the NIH regimen within the previous twelve months. Exclusion criteria: unable to speak English or French, severe cognitive impairment. Participants were identified by health professionals and approached for consent by a research assistant not connected to the patient’s care.

**Fig. 1:**
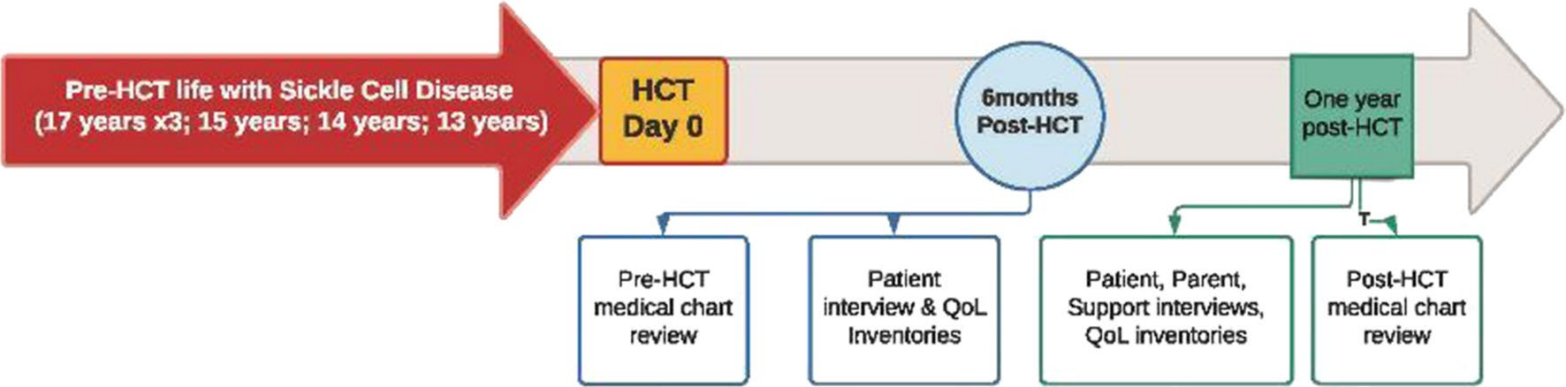
ADaPTS data collection timeline after HSCT

Data sources included: (1) QoL inventories, (2) patient interviews, (3) parent interviews, (4) vital support network (individual(s) identified by the patient who was their confidante during the transplantation process) interview, (5) medical records analysis. The time points were 6 months and 12 months post-transplant for QoL inventories and interview(s) (Fig. 1). All interviews were semi-structured around an interview guide (see “Appendixes A” and “B”).

The PedsQL™ measures the dimensions of health as outlined by the World Health Organization. The inventory was administered twice for the patient and the parent-proxy. Three modules were used the PedsQL™, the PedsQL™ Sickle Cell Disease Module, and the Bone Marrow Transplant Module [17].

Medical charts were reviewed to determine (1) the medical course of the patient over the 12-month period following HCT, (2) major pre-HCT SCD-related complications, (3) any SCD-related and HCT-related complications post-HCT. We also note healthcare providers’ descriptions of the patient’s psychological, social, and spiritual well-being. We analyzed medical records using content analysis approach.

### Data analysis

Data collection and analysis was iterative. Quantitative data was descriptively analyzed and compared to qualitative data. Qualitative data was transcribed verbatim and analyzed. Using Yin’s suggestions on the analysis of multiple case studies, we employed the analytic technique of pattern matching, time series analysis, and cross case synthesis [16, 18]. Step one involved intra-case analysis by assembling evidence within a single case and then analyzing the similarities and differences within the case creating the case description. Step two employed a time series analysis which enabled changes to be tracked in patients QoL. We utilized multiple sources of data to compose a patient timeline and changes across time. In addition, we employed pattern matching as an analytical technique. This enabled examination of patterns across cases. Step three involved cross-case synthesis to synthesize results of the cases.

### Rigor and data quality

Rigor was ensured by following Yin’s suggestions to establish rigor in case studies [16, 18]. With respect to the central construct, we define QoL as “an individual’s perceptions of their position in life in the context of culture, and value systems in which they live and in relation to their goals, expectations, standards and concerns [14].” In line with Yins suggestion, to ensure construct validity, we used multiple sources of evidence [16]. Yin identified that internal validity in case study research is only applicable in causal studies [16, 18]. External validity relates to the generalizability or transferability of the study findings. We achieved external validity by providing verbatim quotes and thick descriptions. Cross-case comparisons with multiple cases strengthens the external validity.

## Results

**Fig. 2:**
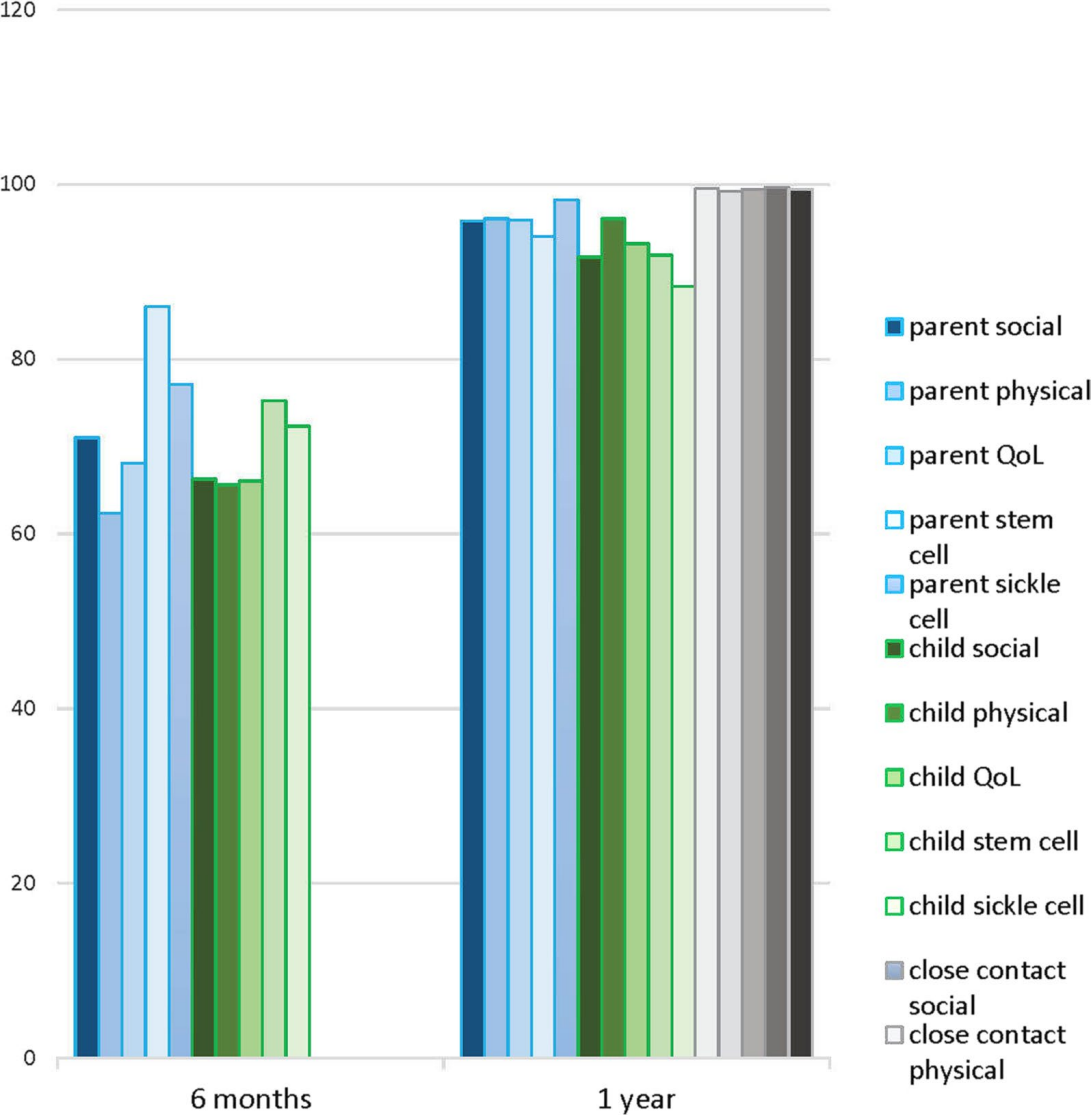
Parent and Adolescent QoL at six months and one year post HSCT as measured by PedsQL Inventories™

A total of 5 participants completed the qualitative interviews. Saturation was achieved. The interviews were conducted in English by an African research assistant at the participants’ home (as per request of the family). All the patients had Sickle Cell Disease Hg SS. The majority were male (80%), and born outside of Canada (80%). Please see Table 1 for demographics. Quality of life data from inventories is combined with interviews. Please see Fig. 2 for mean scores from the inventories between 6 months and 1 year.

**Table 1:** Demographics

Age at HCT	13–18 years (%)	Mean age of 15 years
Male	80	4/5 Participants
Born outside of Canada	80	4/5 Born in either Nigeria, Congo, or Syria
Single parent household	40	2/5 Participants
Genotype HgS	100	5/5 Participants

See Fig. 1 for study timelines.

Medical chart reviews pre-HCT revealed that all patients experienced sickle cell complications which are presented in Table 2.

**Table 2:** Pre-HCT complications

Complication	Participants hospitalized	
	Percentage	Total number (T = 5)
Fever	80	4
Pain	100	5
Acute chest syndrome	40	2

Medical chart reviews post-HCT demonstrated that all 5 patients had successful donor engraftment, achieved donor HbS levels, and none had GVHD. There were no sickle related post-HCT acute events. Four of the six patients had viral infections that were resolved.

Two cases (Patient 1 and 2) are presented below as examples of the data.

### Case one

Patient 1 was born in Africa and diagnosed at one year of life. His pre-HCT life in Africa was characterized by intense physical pain, fevers, and frequent hospitalizations. He appeared physically pale and underweight, with jaundiced eyes and a protuberant stomach. His poor state of health contributed to insomnia, school absenteeism, and physical inactivity. Fear of premature death posed significant psychological stress, which was aggravated in the local community by rumours that portrayed him as “a child on death row.” Stigma from extended family and peers that misrepresented SCD as a contagion accentuated psychological stress, causing him to be socially isolated. During this time, a significant portion of the family income went to paying for healthcare, which brought economic and psychological stress on the family. For this reason, Patient 1 contemplated suicide because he began to view himself as a burden to his family. His parents decided to seek help in Canada, leaving behind their respective successful professional careers.

In Canada, the family experienced downward socioeconomic mobility but were hopeful for a better future for their son. He was hospitalized 5 times in 8 months, prompting consideration of HCT and subsequently transplanted at 17 years of age. While the transplantation went smoothly, the medical preparation was difficult. In the immediate post-HCT period, he experienced several health and social concerns, including hair loss, amnesia, and isolation. He described this as a difficult period that adversely affected his social life. The HCT and its immediate aftermath reactivated thoughts of premature death and doubts about the procedure’s long-term therapeutic benefits. His mother lost her job due to their stay in the hospital. Accordingly, patient 1 and his family described their QoL during the HCT and the period of isolation afterwards to be unsatisfactory.

Patient 1 and his family reported an improved QoL 6 months after HCT. He reported an ability to sleep, walk, and exercise without the usual pain that characterized his pre-HCT life. He described his memory as “weak but improving.” His experience of good health and happiness was greatly enhanced 12 months post-HCT, as his SCD crisis and hospitalizations had ceased. These post-HCT changes have brought about tremendous QoL improvements. When asked whether he would consider undergoing HCT a second time he stated, “Having the transplant is the best decision that my parents ever made.”

### Case two

Patient 2 was born in Canada to African immigrant parents, and was 17 years old at the time of her transplant. As a single parent household, Patient 2’s frequent hospitalizations shifted parental attention away from her younger siblings. She experienced stigma and social exclusion from family in Africa, and was predicted to die prematurely, which resulted in intense sadness for her mother and siblings.

When doctors mentioned HCT during one of her hospitalizations, she resisted and even attempted to dissuade her sister from being the donor. She was frightened of transplant and its side effects, despite a detailed explanation from doctors. Her main concern was the possibility of infertility. Six months post-HCT, Patient 2 and her family reported improvements in her QoL. She could breathe better and perform physical tasks without pain; her eye colour changed; her hair grew back; and she started University. Unfortunately, her menstrual cycle was irregular. Her one-year post-HCT experience was characterized by a crisis-free life, which was capped off by a feeling of psychological relief. Her social life improved as her extended family in Africa initiated relationships with her. Overall, Patient 2 and her family expressed joy at the transplant outcomes, stating that “It was a good decision because my life is changed forever.”

Between the two patients, significant improvements in the physical and psychosocial domains of quality life are apparent. However, these improvements did not fully materialize until a year post HCT.

### Quality of Life by domain

#### Physical QoL

Participants reported a remarkable improvement in their physical QoL, one-year post-HCT.

Prior to undergoing HCT, all the patients experienced pain, fatigue, insomnia, and fevers, which participants termed “SCD crisis.” A parent explained her son’s experiences of physical pain prior to his HCT:

He had pain in his leg, and in his back…all the time, he had pain, pain, pain. And he can’t play.—Mother, Patient 3.

A patient revealed the frequency and severity of her pre-HCT pain:

…it was pretty bad. I would have crisis at least four times a year. Those are crises that I [would] need to go to the hospital for, and not ones that I could control with codeine and stuff at home. I just had a lot more pain.—Patient 2.

The SCD crises often led to hospitalizations. For some patients and their families, these hospital stays were frequent and stressful. A parent explained how her child’s recurrent pre-HCT crises placed their family in a cyclical movement between home and hospital:

As a parent, I saw how he was suffering. We went through a lot of struggle having sickle cell anemia. So it was very hard as a parent…We would have been staying two weeks in the hospital and one week at home—just like that, in and out of the hospital.—Mother, Patient 3.

The fragile nature of the patients’ physical health meant that their ability to perform physical tasks were constrained. Sports activities, such as swimming, and soccer were out of bounds to our participants, although they had the desire to participate.

…because I had sickle cell, there were things like swimming, for example, that I had to get taken out of…All of the time when I would swim in cold water, I would have a crisis.—Patient 2.

A year after the procedure, the experience of pain and fatigue had given way to a crisis-free life, and the patients had the capacity to take up sports.

…when I had sickle cell, I couldn’t play sports. So sometimes when I played sports I get tired quick. Sometimes I will feel pain. But after the transplant, it is okay. I can now play sports with my friends, like soccer, basketball. I don’t really feel that pain [and] all the tiredness [and] all the fatigue.—Patient 1.

All 5 patients physical QoL improved one-year post-transplant as per their interviews.

#### Social QoL

The pre-HCT social QoL of the patients and their families was poor, and typically characterized by stigma, social isolation, and parental absenteeism from work. All patients experienced stigma prior to undergoing HCT. A parent revealed how such perceptions caused her daughter to struggle with gaining acceptance and a sense of belonging in their extended family:

Back in Nigeria they just think that if you have sickle cell you don’t live long. Your life span is very short. So, people don’t even count them among the children, because they feel that maybe this child will die soon…my mother-in-law [said] F. is not a child. I shouldn’t count F. as a child because she has sickle cell.—Mother, Patient 2.

Another parent revealed how her son’s SCD status was stigmatized each time his illness was revealed to members of their community.

When I tell them oh he has sickle cell, they look at him differently, as if to say, “don’t go close to my daughter…I don’t want you liking my daughter for marriage.” And all that. And they look at him as if it is a disease that is transferrable.—Mother, Patient 1.

Pre-HCT social activities with peers were significantly curtailed by the SCD. All patients revealed how fatigue and the potential for a pain crisis caused them to refrain from activities, leaving them socially isolated and disconnected. Patient 4 revealed how the disease affected his social activities and interactions.

Like before I would go so tired before with my friends. I can’t hang out with them. I can’t play with them, anything, I can’t go with them. Sometimes at class I can’t go at lunch or play.—Patient 4.

The HCT process, the stay in hospital, and the need to isolate post-HCT at home (a period ranging from 3 to 6 months was necessary to avoid opportunistic infections) were equally isolating. Patients described this isolation as a difficult period.

I was bored every time. Because I was stuck inside [the hospital] mostly…because during that time, my immune system was low, so I was like very easily get infected.—Patient 3.

Academic studies were interrupted and described as negatively affecting social QoL.

I was missing school. I was kind of thinking like how I could catch up and like what the problem would be if I come late, because I know that I will have a pile of work coming to me…Because the school is like very serious. And they have a lot of things going on like, tests, quizzes.—Patient 5.

In one single parent family with multiple children, parental absence from home during pre-HCT periods of hospitalization affected the fulfilment of the emotional and material needs of the other children. A sibling explained how frequent pre-HCT hospitalizations affected their wellbeing:

It was hard for us, like you know, when we needed things at home. Mom wasn’t there because she would be with my sister in the hospital…For school I would need my mom to sign something, and then she wasn’t there to do that. Sister, Patient 2.

The absence of parents from work and its financial consequence during times of hospitalization constituted a source of socioeconomic disadvantage. A sibling of Patient 2 stated, “[Mom] had to take leave off work…My brother couldn’t always get the things he needed because my mom wasn’t getting pay.”

Although unintended, such parental absence can have negative consequences for social relationships within families, more so when parental absence is frequent, extensive, and sustained at the expense of the emotional development and material wellbeing of other family members.

Post-HCT, the adolescents gained social acceptance in their larger families and communities that once stigmatized and excluded them. One patient explained how the transplant has helped to mend her relationship with her extended family:

Some of my family members didn’t really accept me having sickle cell and stuff like that. And so like now that I don’t have sickle cell, some of those family members have tried to come in contact with me and talk to me now.—Patient 2.

A year after undergoing HCT, all patients experienced improvements in their social relations from all sources of data collected.

### Psychological QoL

The pre-HCT life experiences of the patients were overshadowed by sadness, dissatisfaction with life, and psychological stress. The majority of patients commented that their future was bleak and may lead to premature death. Patient 1 revealed how his pre-HCT health conditions affected his thoughts:

I thought I would maybe die someday. That is something I would always think in my head. In Nigeria I always thought I was going to die, because I was always sick. So I thought the future was going to be very bad.

Negative thoughts and the pain experienced during SCD crises created despair, some patients had cried frequently at the thought of knowing that their illness was a lifetime condition. The feeling of sadness and despair often extended to the entire family. A parent explained how the pre-HCT experience created an atmosphere of sadness in the household:

I think that another challenge with the kids, you know, if one of them is not feeling well, so you see all of them look like they are sad. That affects also parents, when you see your kids sad, [and] they are not happy as you want them to be. So, it affects yourself too. Father, Patient 3.

Fear of the unknown was a constant part of the life of parents with children ailing from SCD. Parents felt a need physically be with the patient. The mother of patient 4 recollected that “*his dad [was] always thinking [at work – what happens with my son*?” The thought of having a permanently ailing child was a source of psychological burden to parents.

Although most patients and their families were relieved after learning about HCT, anticipation of HCT and the potential negative outcomes, including death and infertility, produced additional psychological stress. Information sessions provided included reference to the possibility of adverse outcomes and for some were a source fear and anxiety.

I did not know what to expect. I was dreaming even that E. died. Sometimes I would dream that he died during the program. So I was always afraid until that day. Mother, Patient 1

Patient 5 added, “*I was kind of having second thoughts. Because I kind of thought that was a lot to handle*.”

The patients and their families called for pre-transplantation information and counselling sessions that are encouraging and accurate.

A year after the transplant, the psychological stress diminished and for some families was eliminated. All 5 patients and their families expressed a feeling of relief, happiness, and hope for the future. The father of Patient 3 explained how HCT has brought happiness and a sense of psychological relief to his entire family:

When we started seeing the outcome…I can say our mind changed. We saw that our mind can be cooled down, compared to before the transplant…because there is no more crisis… He is doing sports.

Patient 4 added to this general sense of relief, “*I am happy now. I don’t know how to say, but it is very good*.” These narratives together convey a general sense that HCT has yielded positive psychological outcomes for both patients and their families (Table 3).

**Table 3:** Coding summary: changes in experiences and QoL

QoL domains	Pre-HCT	HCT and immediate aftermath	6 months post-HCT	12 months post-HCT
Physical domain	Patients experienced intense pain and frequent hospitalizations Patients looked physically pale and sickly (underweight, red eyes, yellow eyes, protruded stomach, etc.) Patients experienced shortness of breath Patients experienced low energy Patients were often tired and had to avoid strenuous physical activities (e.g., soccer, running, swimming, etc.) Low blood Bled frequently through the nose Depended on lots of medication to treat pain, infections, low blood count, etc	Low energy and tiredness persisted Hair loss due to chemotherapy intensified Bedridden for weeks after HCT Patients experienced fever and vomiting Patients were prone to infections Medications dependence persisted Patients gained weight	Patients experienced no more pain No hospitalizations Patients could sleep better Patients could breathe better Patients could eat better Hair grew back Patients gained energy and could undertake physical exercise (e.g., running) Everything felt a lot easier Still had frequent doctor’s appointments	Patient experienced no pain Patients experienced no crisis and hospitalizations Patients experienced no shortness of breath Patients could sleep much better Blood levels had significantly increased Medication-dependence reduced dramatically Patients looked physically grown, both in weight and height Patients gained significant amounts of energy and could play sports
Social domain and supports	Patients experienced stigma and isolation Society perceived SCD as a curse Patients had limited interaction and socialization (e.g., play, social events, etc.) with peers due to fear of sudden illness Parental absenteeism due to long hospital stays Disagreement between parents and patients over medication regimen and the decision to undergo transplantation	Patients and siblings lost friends due to long hospital and home stays post-HCT Patients stayed away from school and friends to recuperate Received visitation from church members and family friends during post-HCT hospital stays	Patients went out more frequently and socialized more with friends	Interacted more with family members back home in Africa Patients returned to school
Economic domain	Parents spent lots of money on hospital bills and medication Parents missed work and income earning opportunities due to long hospital strays Parents experienced financial problems due missed work and high medication expenditures	Parents did not pay for the HCT procedure but had to pay for required medications Parents quit job due to long hospital stays Parents experienced financial problems due to loss of employment and increased medication expenditures		
Psychological domain	Patients expected a shorter lifespan Patient feared they would suddenly die Patients and parents were scared of possible infertility due to radiation and chemotherapy treatments Patients feared they would die during transplantation process Patients were worried about missing school classes after transplantation Parents and siblings were sad and mentally stressed	Patients and parents feared that the HCT procedure would fail Patients were concerned about missing school and doing more work later to catch up with their studies	Patients and parents were happier Patients and parents’ mental health improved Patients had thoughts of premature menopause and infertility due to radiation and chemotherapy treatments Patients could focus more in school	Patients felt they truly overcame sickle cell disease Patients felt their life was as normal as everyone’s Patients felt satisfied with life Parents and patients felt mentally relieved and burden-free

## Discussion

Our study contributes a qualitative dimension to the nascent but growing research on the relationship between HCT and QoL of pediatric patients with SCD. We investigated and compared the physical, social, and psychological QoL of patients and their families before and after HCT. We found the post-HCT experiences were overwhelmingly positive. QoL in all domains improved one-year post-HCT. In the physical domain, patients reported a change from multiple episodes of SCD crisis and hospitalization at baseline to a crises-free life one-year post-HCT. In addition, unlike their pre-HCT experiences, patients reported no episodes of fatigue, yellowed eyes, or shortness of breath post-HCT. Consequently, the patients participated in strenuous physical activities, such as running, swimming, and walking.

In the social domain, our participants reported a life that has changed from frequent experiences of stigmatization and social isolation to one in which they felt more socially accepted by the same extended families and communities that once excluded them. Following their respective successful procedures, 3/5 patients were able to reconnect with their extended families and ethnic communities in ways that demonstrated respect and acceptance. A greater participation in social activities post-HCT improved life. In the psychological domain, the patients and their families expressed happiness, greater satisfaction with life, and a sense of relief. However, we will note that the post-HCT improvements in the QoL in our participants did not follow the linear improvements previously reported for this demographic [11]. Along with Shenoy et al. [19], our results demonstrate that the QoL of pediatric patients following HCT is one that oscillates between decline and baseline levels before improving. Indeed, the QoL of our participants was reported to be unsatisfactory during the HCT process and the period following discharge from hospital. After, QoL gradually improved at one-year post-HCT compared to 6 months post HCT. We argue here that the pre-HCT conditioning regimens, the medications required, and social isolation were factors that influenced our participants’ initial reports of unsatisfactory QoL, especially in the short period before and after the HCT.

The improvements in one-year post-HCT reported among our participants could be attributed to the success of the HCT itself. None of our participants experienced adverse outcomes, such as graft rejection, sickling crises (including pain crises), or graft‐versus‐host disease (GVHD)- complications observed in SCD patients undergoing HCT in other cohorts [20–22]. All 5 patients received non-myeloablative HCT from sibling donors; matched sibling donor outcomes are superior to alternative donor HCT, and GVHD and rejection free survival outcomes with the NIH regimen in adolescents and young adults are better than those described with other regimens better outcomes than other types of transplants described [12, 23–25]. Post-transplant the participants QoL gradually improved in comparison to their lived experience with sickle cell disease. By initially affecting patients’ physical QoL, an event-free life also triggered positive changes to social and psychological aspects of life, given the interconnected nature of all 3 domains. For example, by alleviating physical pain, HCT affects social aspects of life by engendering participation in peer activities.

Our participants’ report of improved QoL post-HCT compares favorably with, but differs from the findings of other studies. Research conducted in the United States and Canada, indicates post-HCT improvements in the physical, social, and psychological domains of QoL for both patients and parents [13, 26, 27]. In Bhatia et al.’s study [13] involving self-reported measures of QoL, the overall health-related QoL of patients, measured on a scale of 0–100, jumped from 66.1 pre-transplant to 82.3 one-year post-transplant. However, despite reporting predominantly positive post-transplant outcomes [28], Panepinto et al. [22] found 17/54 HCT recipients experienced reoccurrence of sickle cell symptoms due to GVHD related complications and Gluckman et al. reported event free survival was 93% and a 5-year probability of GVHD-free survival of 86% for patients younger than 16 years [23]. A recent systematic review reported that participants with SCD demonstrated improved quality of life in all QoL domains (as measured by QoL inventories) [28]. In comparison, our study explores the depth of impact in multiple domains of QoL that has not been highlighted in the prior studies.

The result on post-HCT QoL was a similar or improved status from baseline (life with Sickle Cell Disease) QoL. The difference between previously published work and our study may be due to the lower intensity conditioning regimen and the lack of GVHD or rejection in our cohort [12]. Although HCT was overwhelmingly positive it is important to continue to follow-up and evaluate QoL status until full recovery is achieved. This is necessary in light of some research suggesting long-term complications with other HCT regimes [22, 24, 29].

Despite well defined medical and clinical outcomes, concern exists around the psychosocial impact of the HCT. Several studies [30, 31] have reported post-HCT psychosocial stress among parents and patients over concerns of potential graft failure, sexual dysfunction, and infertility. In addition to these concerns, we found anxiety relating to hair loss, cognition difficulties, and menstrual irregularities post-HCT. These outstanding concerns merit investigation, and certainly can be the focus of pre-HCT counselling and education sharing throughout the HCT journey. These results clearly identify an opportunity to improve the provision of accurate and accessible HCT educational materials for patients and families with SCD. On the social side, the intensity of parental care required during the first 6 months post-HCT led to parental absenteeism from family and work. This absence produced unintended social outcomes, including income losses and material deprivation, although this absenteeism and its social impacts were not any worse than they were during pre-HCT hospitalizations. There is a dire need for more financial supports for patients and families with SCD, including those undergoing HCT. Resources for HCT recipients with non-malignant diagnoses should be comparable to those offered to children and adolescents undergoing HCT as cancer therapy.

Our findings have implications for policy, research, and practice. The study confirms the eventual positive impact of HCT on QoL for adolescents with SCD. There is need for health policies that expand access to the therapy for pediatric patients across Canada and the world. The current practice in Alberta is to offer the therapy to patients with SCD and an available ABO compatible (or minor incompatible) HLA matched sibling donor. However, the current need for a matched sibling donor means that curative therapy is offered to a small proportion of patients. Advances in alternative donor HCT and gene therapy are needed with a goal to offer safe cure to every patient with SCD. Patients and families are seeking information that is both reassuring and accurately describes risks. This information and referral for consideration of HCT requires communication and collaboration between hematologists, transplant physicians, and patients/families [32]. While some families may receive this information and decide that HCT is not the right choice for them, it is noteworthy that some of our patients contemplated withdrawing from HCT due to information that did not realistically prepare them for the experience. Such information must accurately describe the anticipated physical and psychological complications post-HCT, provide realistic expectations for pain outcomes and define success in the context of possible irreversible and established organ damage and chronic pain syndromes. The findings of this research can be used to refine education sessions, which should be iterative throughout the HCT trajectory. Given that the demographic is predominantly African, information sharing and counselling sessions should include a cultural dimension. Access to resources such as the National Marrow Donor Program’s Peer Connect Program are important. The Sickle Cell Transplant Advocacy and Research Alliance aims to improve HCT education provided to all patients and families (www.curesicklenow.org).

Our study has a number of strengths. The mixed methodology lends a unique and in-depth analysis of the patient’s quality of life during their stem cell transplant journey [14–18]. Second, the use of multiple viewpoints builds a robust impression of the patient’s quality of life. Third, the transplant regime was constant for all study participants allowing for comparison across individuals. Finally, individuals from the study team are African immigrants including the researchers conducting the interviews and performing the data analysis thereby allowing for a deeper understanding of the cultural aspects that were described.

There is a need for further quality of life research after HSCT for sickle cell disease to help inform families, providers and patients. Patient (age, gender, etc.) characteristics, HSCT donor, HSCT regime require further investigation to create the body of literature to inform choices and weigh risk:benefit ratio of treatment. Long term data is also critical to inform the field about the durability of the results over time.

## Conclusions

Sickle Cell Disease is a chronic debilitating and life limiting disease with multiple sociopsychological concerns. Select SCD patients now have the opportunity to undertake a promising novel curative therapy (minimal conditioning hematopoietic cell transplant). However, there are numerous unknowns and the HCT is not without risk. This study is the first to provide an in-depth analysis of the adolescent patient’s quality of life after HCT for SCD. The results strongly support HCT and patients, parents, and support networks speak to the incredible benefits in physical, social and psychological quality of life domains. Families called for pre transplant meetings to convey the benefit and not overemphasize the potential negative effects of HCT as they found information sessions to induce anxiety and fear.

## Data Availability

Transcripts cannot be provided due to the ability to identify an individual participant. However, medical chart reviews, Interview scripts and more are available upon request.

## Appendix A

### Changes in experiences and QoL

**Table:**

QoL domains	Pre-BMT	BMT and immediate aftermath	6 months post-BMT	12 months post-BMT
Physical domain	Patients experienced intense pain and frequent hospitalizations Patients looked physically pale and sickly (underweight, red eyes, yellow eyes, protruded stomach, etc.) Patients experienced shortness of breath Patients experienced low energy Patients were often tired and had to avoid strenuous physical activities (e.g., soccer, running, swimming, etc.) Low blood Bled frequently through the nose Depended on lots of medication to treat pain, infections, low blood count, etc	Low energy and tiredness persisted Hair loss due to chemotherapy intensified Bedridden for weeks after HCT Patients experienced fever and vomiting Patients were prone to infections Medications dependence persisted Patients gained weight	Patients experienced no more pain No hospitalizations Patients could sleep better Patients could breathe better Patients could eat better Hair grew back Patients gained energy and could undertake physical exercise (e.g., running) Everything felt a lot easier Still had frequent doctor’s appointments	Patient experienced no pain Patients experienced no crisis and hospitalizations Patients experienced no shortness of breath Patients could sleep much better Blood levels had significantly increased Medication-dependence reduced dramatically Patients looked physically grown, both in weight and height Patients gained significant amounts of energy and could play sports
Social domain and supports	Patients experienced stigma and isolation Society perceived SCD as a curse Patients had limited interaction and socialization (e.g., play, social events, etc.) with peers due to fear of sudden illness Parental absenteeism due to long hospital stays Disagreement between parents and patients over medication regimen and the decision to undergo transplantation	Patients and siblings lost friends due to long hospital and home stays post-HCT Patients stayed away from school and friends to recuperate Received visitation from church members and family friends during post-HCT hospital stays	Patients went out more frequently and socialized more with friends	Interacted more with family members back home in Africa Patients returned to school
Economic domain	Parents spent lots of money on hospital bills and medication Parents missed work and income earning opportunities due to long hospital strays Parents experienced financial problems due missed work and high medication expenditures	Parents did not pay for the HCT procedure but had to pay for required medications Parents quit job due to long hospital stays Parents experienced financial problems due to loss of employment and increased medication expenditures		
Psychological domain	Patients expected a shorter lifespan Patient feared they would suddenly die Patients and parents were scared of possible infertility due to radiation and chemotherapy treatments Patients feared they would die during transplantation process Patients were worried about missing school classes after transplantation Parents and siblings were sad and mentally stressed	Patients and parents feared that the HCT procedure would fail Patients were concerned about missing school and doing more work later to catch up with their studies	Patients and parents were happier Patients and parents’ mental health improved Patients had thoughts of premature menopause and infertility due to radiation and chemotherapy treatments Patients could focus more in school	Patients felt they truly overcame sickle cell disease Patients felt their life was as normal as everyone’s Patients felt satisfied with life Parents and patients felt mentally relieved and burden-free

## Appendix B

### Interview guide: patient at 6 months

Thank you for agreeing to be interviewed. There are four parts to the interview questions. First, we will discuss your experience before bone marrow transplant. Second, we will discuss your experience during BMT and hospitalization. Third, we will discuss your experience in the first six months after BMT. Lastly, we will discuss any recommendations you have for other patients, health professionals and policy makers.

#### Pre-BMT

- What was your experience as a child or adolescent with SCD before BMT?
- How was your health like before you had BMT? (Probe physical, mental, social and psychological)
- What social, economic or cultural challenges related to SCD or BMT did you face?
- How did this challenges (including health) affect your happiness and quality of life pre-BMT?
- How satisfied were you with your health and your life before you had BMT?
- What motivated you to get a BMT?
  - How did you arrive at the decision to get BMT?

#### During BMT

- Tell me about your experience during the BMT?
- What health challenges did you face? (Probe physical, mental, social and psychological)
- What social, economic or cultural challenges related to SCD or BMT did you face?
- How did this challenges (including health) affect your happiness or quality of life?
- What was your experience with the healthcare system and health care professionals?
- What or who was most helpful to you during the BMT?
- Was your experience during BMT and hospitalization what you expected?
  - If yes, how so?
  - If no, what expectations did you have about BMT that was not met?
- What would have been helpful for you to know prior to your BMT and hospitalization?

#### After BMT (first 6 months)

- Tell me about your experience after BMT (first six months)?
- What health challenges did you face? (Probe physical, mental, social and psychological)
- What social, economic or cultural challenges related to SCD or BMT did you face?
- How happy and satisfied with life are you now after your BMT? (Probe into each of the sections that are rated poorly on the quality of life survey)
- What or who has been helpful or supportive to you in this first six months after BMT?
- Looking back to when you and/or your family made a decision to get BMT, do you think you and/or your family made a good decision?
  - Would you make the decision again if you were in the same situation? Please explain why or why not

#### Implications

- What would you tell other patients with SCD about BMT?
- What would you like healthcare professionals to know about caring for patients with SCD or those getting treatment for BMT?
- What would you like decision makers and policy maker to know about health services for patients with SCD or those getting treatment for BMT?

### Interview guide: patient at 12 months

Thank you for agreeing to be interviewed again. For this interview, we will discuss your experience in the last six months (or since our last interview). Next, we will discuss your response to the quality of life inventory. We will also discuss any implications for health care providers and policy makers.

#### Post BMT: 6 months to 12 months

- What has been your experience since the last time you had an interview?
- How has your health been like since the last time you had an interview?
  - Probe for health events based on chart audit
  - Probe physical, mental, social, spiritual and psychological health
- What social, cultural and economic challenges related to SCD or BMT have you faced since the last interview?
- How did this challenges (including health) affect your happiness and quality of life?
- How happy and satisfied with life are you now, approximately 12 months after the BMT?
- Looking back to when you and/or your family made a decision to get BMT, do you think you and/or your family made a good decision?
  - Would you make the decision again if you were in the same situation? Please explain why or why not

#### Clarifying previous data

- Probe into results of any data on the quality of life inventory in-which patients do not have the highest level of quality of life?
- Probe into any previous data gathered from diverse sources that may be unclear or inconsistent

#### Implications

Based on your experience in your first year after BMT:

- What would you tell other patients with SCD about BMT?
  - Would you advice patients with SCD to get BMT or not? Why or why not?
- What would you like healthcare professionals to know about caring for patients with SCD who are considering treatment for BMT?
- What would you like healthcare professional to know about caring for patients with SCD who are getting BMT?
- What would you like healthcare professionals to know about caring for patients after they have had a BMT?
- What would you like decision makers and policy maker to know health services for patients with SCD or those who have received treatment for BMT?

### Interview guide: parents and support system

Thank you for agreeing to be interviewed. There are four parts to the interview questions. First, we will discuss your child, friend etc. experience before bone marrow transplant. Second, we will discuss his/her experience during BMT and hospitalization. Third, we will discuss his/her experience in the first year after BMT. Lastly, we will discuss any recommendations you have for other patients, parents, health professionals and policy makers.

#### Pre-BMT

- What was the experience of your child, friend etc. with SCD before BMT?
- How was life like for the family before BMT?
- What social, economic or cultural challenges related to SCD or BMT did the family face?
- How did this challenges (including health) affect the child’s happiness and quality of life pre-BMT?
- How satisfied were you with the child’s health and life before you had BMT?
  - Were you supportive of the decision to get BMT? Please expand on why or why not?
  - For parents: How did the family arrive at the decision to get BMT?

#### During BMT

- Tell me about the child and families experience during the BMT?
- What health challenges did the child face?
- What social, economic or cultural challenges related to SCD or BMT did the family face?
- How did this challenges (including health) affect the child’s happiness or quality of life?
- What was your experience with the healthcare system and health care professionals?
- For Parents: What or who was most helpful to you during the BMT?
- Was the child and family’s experience during BMT and hospitalization what you expected?
  - If yes, how so?
  - If no, what expectations did you have about BMT that was not met?
- What would have been helpful for you to know prior to the BMT and hospitalization?

#### After BMT (first year)

- Tell me about the experience after BMT?
- What health, social, economic or cultural challenges related to SCD or BMT did the family face?
- What changes (if any) have you notice in the child and in your family in the first year after BMT?
- What or who has been helpful or supportive to you and the child in this first year after BMT?
- Looking back to when the family made a decision about BMT, do you think the family made a good decision?
  - Would you make the decision again if you were in the same situation? Please explain why or why not

#### Implications

- What would you tell other families with a child with SCD about BMT?
- What would you like healthcare professionals to know about caring for patients with SCD who are considering or getting treatment for BMT as well as their families?
- What would you like decision makers and policy maker to know health services for patients with SCD or those getting treatment for BMT as well as their families?

## Abbreviations

HCT: Hematopoietic cell transplantation
SCD: Sickle cell disease
QoL: Quality of life
